# The Moderation of Self‐Compassion Between the Relationship of Compassion Competence and Caring Behavior

**DOI:** 10.1155/jonm/3095657

**Published:** 2026-03-18

**Authors:** Meaad T. Alanazi, Manal F. Alharbi

**Affiliations:** ^1^ Innovation Center, Ministry of Health, Riyadh, Saudi Arabia, behdasht.gov.ir; ^2^ Department of Maternal and Child Health, College of Nursing, King Saud University, Riyadh, Saudi Arabia, ksu.edu.sa

**Keywords:** caring behavior, compassion competence, pediatric registered nurses, Saudi Arabia, self‐compassion

## Abstract

**Background:**

Understanding factors influencing caring behavior and compassion competence is crucial for delivering compassionate nursing care. Recent research suggests that self‐compassion significantly affects both caring behavior and compassion competence.

**Purpose:**

The study aims to investigate the moderating effect of self‐compassion on pediatric nurses’ caring behavior and compassion competence in Riyadh, Saudi Arabia.

**Methods:**

This study employed a nonexperimental quantitative design with a descriptive cross‐sectional approach, involving 202 pediatric nurses. Data were gathered through a four‐part structured self‐administered questionnaire, which included demographic information, a compassion competence scale, a caring behavior inventory (16‐item version), and a self‐compassion scale.

**Results:**

The majority of participants were female (97%) and aged 31–40 years (45.5%), with 6–10 years of experience (38.6%). Self‐compassion scores averaged 3.48 (SD = 0.52), compassion competence averaged 4.06 (SD = 0.59), and caring behavior averaged 86.33 (SD = 10.31). A moderate positive correlation was found between self‐compassion and compassion competence (*r* = 0.285, *p* < 0.001), a weak positive correlation was found between self‐compassion and caring behavior (*r* = 0.183, *p* = 0.009), and a moderate positive correlation was found between compassion competence and caring behavior (*r* = 0.397, *p* < 0.001).

**Conclusion:**

This study reveals that pediatric registered nurses exhibit high levels of compassion, competence, and caring behavior. However, self‐compassion does not significantly influence the relationship between these two factors. The findings offer a framework for further exploration of the influences on caring behavior and compassion competence among pediatric nurses, aiding in the development of policies and interventions to enhance pediatric patient care.

## 1. Background of the Research Study

Caring and compassion are central to contemporary nursing practice, particularly in high‐demand clinical environments where nurses must balance technical responsibilities with emotional and relational aspects of care [1, 2]. Compassionate care requires nurses to recognize patients’ needs, respond empathetically, and build therapeutic relationships that enhance safety, comfort, and communication [3, 4]. Recent research highlights that nurses’ caring behaviors are influenced by emotional awareness, empathy, and the ability to remain attuned to patients’ experiences [1, 5]. Self‐compassion has also emerged as a relevant factor, as it supports emotional regulation, enhances empathy, and contributes to nurses’ capacity to deliver compassionate care [6, 7]. Although the international literature has explored caring behavior, compassion competence, and self‐compassion across various nursing contexts, there is limited evidence regarding the specific relationships among these constructs within the Saudi Arabian setting, especially in pediatric care. Recent regional research indicates that Saudi pediatric nurses encounter distinct communication challenges, emotional burdens, and family‐centered care requirements that differ significantly from those faced by adult nurses [8, 9]. Nonetheless, much of the existing regional research has predominantly focused on general nursing populations rather than pediatric nurses. It has failed to examine how psychological resources, such as self‐compassion, may affect professional behavior. Crucially, few studies have investigated whether self‐compassion moderates the association between compassion competence and caring behavior, despite evidence highlighting the importance of both constructs in managing emotional labor and maintaining care quality [10, 11]. Consequently, how self‐compassion influences pediatric nurses’ caring practices within the Saudi healthcare context remains relatively unexplored. This highlights the need for a context‐specific investigation to better understand how these interconnected constructs operate within a population facing considerable emotional, relational, and cultural challenges.

## 2. Literature Review

Compassionate and caring conduct is central to high‐quality nursing practice, particularly in pediatric environments where nurses must address the physical, emotional, and developmental needs of children and their families. Pediatric nurses encounter distinct challenges, including communication difficulties, emotionally intense interactions with parents, ethically complex situations, and frequent exposure to patient suffering. These demands underscore the essential role of compassion competence, defined as the nurse’s ability to recognize distress, respond empathetically, and deliver patient‐centered care. Although compassion, competence, and caring behaviors have been extensively examined worldwide, the emotional labor intrinsic to pediatric nursing underscores the need for additional internal resources, particularly self‐compassion.

Self‐compassion characterized by self‐kindness, mindfulness, and awareness of shared humanity has been identified as a significant psychological resource for improving nurses’ emotional regulation and well‐being. Contemporary evidence shows that nurses with higher self‐compassion report better sleep quality, greater resilience, and lower burnout, particularly in emotionally demanding specialties such as pediatrics [12–14]. Self‐compassion also enhances professional quality of life by increasing compassion satisfaction and buffering against compassion fatigue, which is highly relevant for pediatric nurses navigating complex family dynamics and emotionally charged clinical encounters [15]. Nurse–parent interactions—an essential component of pediatric care—also benefit from nurses’ self‐compassion, as empathy and emotional presence strengthen relational trust and communication [16]. Collectively, these findings highlight self‐compassion as a meaningful factor influencing both caring behaviors and relational competence.

Compassion competence itself remains fundamental to high‐quality pediatric nursing. Recent frameworks conceptualize compassion as a multidimensional construct that involves sensitivity to patient suffering, empathetic understanding, and a commitment to alleviating distress [2–4]. Higher compassion competence has been associated with improved clinical performance, increased job satisfaction, and reduced emotional burden [10, 17]. However, compassion competence is frequently challenged in high‐stress pediatric settings, such as intensive care and oncology units, where heavy workloads, complex emotional demands, and repeated exposure to suffering elevate the risk of burnout and compassion fatigue [18, 19]. Understanding how personal psychological resources—particularly self‐compassion—interact with compassion competence is therefore vital for sustaining caring behaviors.

Caring behaviors, expressed through both technical care and emotional support, are central indicators of nursing quality and patient satisfaction. Recent evidence shows that caring behaviors enhance patient recovery, communication, and trust, whereas inadequate caring may adversely affect the quality of care [20, 21]. Pediatric nurses must continuously balance technical interventions with emotional support for children and their families, making their caring behaviors particularly sensitive to stress, emotional exhaustion, and workplace conditions [22, 23]. These findings reinforce the importance of considering both professional competencies and individual psychological factors in explaining caring behaviors.

Emerging evidence indicates that compassion competence, self‐compassion, and caring behaviors are interconnected, with mindfulness and emotional awareness playing central roles in strengthening caregiving capacity [24, 25]. Self‐compassion may enhance caring behaviors by promoting resilience, empathy, and reflective practice [26, 27]. Pediatric nurses with higher professional quality of life also demonstrate stronger communication skills and more humanistic caregiving behaviors, emphasizing the role of emotional well‐being in sustaining compassionate care [28]. Together, this growing body of research suggests that both self‐compassion and compassion competence meaningfully contribute to caring behavior; however, their combined influence—particularly the moderating role of self‐compassion—remains insufficiently examined in pediatric nursing contexts. This gap underscores the need for empirical studies exploring how these constructs interact in pediatric settings where emotional demands and relational complexity shape the delivery of compassionate care.

Despite extensive global research, significant knowledge gaps remain in the Middle East and Saudi Arabia. The regional literature highlights high emotional demands among pediatric nurses, compassion fatigue, culturally influenced communication challenges, and the influence of expatriate workforce dynamics [8, 9]. However, studies have not evaluated whether self‐compassion functions as a moderator between compassion competence and caring behavior. Furthermore, although compassion, competence, and caring behaviors have been explored separately in some Gulf‐region studies, no prior research has examined their interrelationships in pediatric nursing in Saudi Arabia.

Two recent Middle Eastern contributions highlight the importance of examining contextual, cultural, and emotional dimensions of nursing care [29, 30]. Building on these, a focused exploration of the role of self‐compassion is warranted, particularly given the cultural norms surrounding empathy, caregiving expectations, emotional expression, and professional dynamics in Saudi Arabia. Therefore, this study addresses a critical gap by investigating

1. The levels of self‐compassion, compassion competence, and caring behavior among pediatric nurses.
2. The relationships between these variables.
3. Whether self‐compassion moderates the relationship between compassion, competence, and caring behavior.

Understanding this moderating effect offers practical implications for strengthening pediatric nursing practice, improving emotional resilience, and enhancing patient and family care outcomes within the Saudi healthcare context.

### 2.1. Conceptual Framework

**Figure 1:**
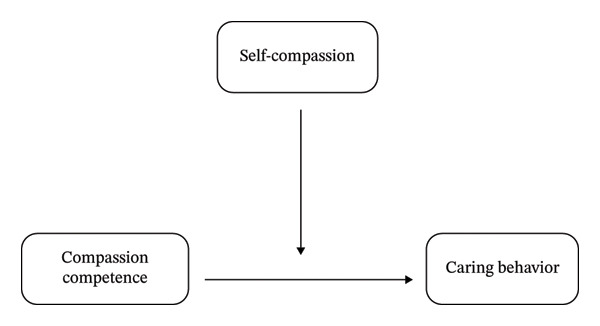
The conceptual framework of the study. *Note:* In this framework, self‐compassion is suggested to act as a moderating factor that influences the connection between compassion competence and caring behavior.

Watson’s human caring theory is an appropriate framework for this study for several key reasons. First, it emphasizes the integral aspects of human beings and their physical, mental, and emotional well‐being. Second, it asserts that love is fundamental to nursing care and healing. Third, the theory outlines three essential elements of nursing care: human interactions, interpersonal relationships, and an awareness of the healing process. Fourth, it provides a practical framework for clinical application through 10 Caritas processes: these include practicing kindness toward oneself and others, being authentically present, developing spiritual practices, establishing caring relationships, expressing empathy and forgiveness, utilizing all means of knowing, engaging in teaching and learning, and fostering a caring environment that celebrates life’s miracles [31, 32]. Incorporating this theory into research underscores the significance of caring relationships and well‐being. This study will explore how self‐compassion moderates the relationship between compassion competence and caring behavior among pediatric nurses (see Figure 1).

## 3. Methods

This study employed a nonexperimental quantitative research design to address the research question. This method is suitable for nursing research as it minimizes bias and examines variables in their natural state without intervention [33]. A descriptive cross‐sectional approach assessed the impact of self‐compassion on caring behavior and compassion competence among pediatric nurses, aligning with the survey’s aim to capture a specific point in time. This approach is also time‐efficient and cost‐effective.

### 3.1. Setting and Sample

The study was conducted at King Khalid University Hospital (KKUH), a facility providing comprehensive medical services, addressing primary, secondary, and tertiary healthcare needs. The target population comprises pediatric nurses from diverse units, reflecting varying experience levels and nationalities, making KKUH an ideal research setting. KKUH is accredited by the Saudi Central Board for Accreditation of Healthcare Institutions, ensuring high healthcare standards and serving the pediatric population in Riyadh city. Initially, the research aimed to identify the target population for participant recruitment, comprising 276 pediatric nurses. Inclusion criteria required registered nurses (RNs) in pediatric departments who communicated effectively in English, while excluding nonparticipating nurses and interns. A cross‐sectional study design necessitated a specific sampling formula [34]. The Taro Yamane method [35] was utilized to determine the sample size. With a 95% confidence level (*z*‐score = 1.96) and a 5% margin of error, the calculated sample size was 164, augmented by 10% to account for potential missing data, resulting in a final sample of 181 pediatric RNs. This robust sample supports reliable statistical analysis and the generalizability of the results. The study employs controlled quota sampling, which integrates a nonprobability sampling approach to systematically manage data collection outside a random framework. This method facilitates selecting a predetermined number of participants from each pediatric department, thereby obtaining results that more accurately reflect the intended sample [33]. The controlled quota technique for participant selection in the study is outlined as follows. Firstly, a hard copy of the questionnaire was distributed to all selected participants. Also, as facilitated by the hospital’s internal communication department, the online questionnaire was emailed to all pediatric nurses in different positions. Subsequently, participants were stratified by hospital department, including the Pediatric Intensive Care Unit (PICU), Neonatal Intensive Care Unit (NICU), pediatric clinic, surgical and medical pediatric wards, and the Emergency Room (ER). This step involved identifying the necessary number of participants required from each stratum. The next step was to determine the quotas for each stratum within the target population. Finally, the sample size was refined based on the established proportions for each stratum.

### 3.2. Data Collection Procedure

Permission and informed consent were obtained from the relevant departments and volunteers before data collection. The questionnaire was distributed only after receiving the necessary approvals. Coordination with department supervisors ensured optimal timing for participation. An email invitation was sent to pediatric RNs, including a link to the Google form questionnaire with an informed consent section stating that by proceeding, they agree to participate, as this is considered their written informed consent. Responses were submitted anonymously to maintain confidentiality, with participants informed of the voluntary nature of their participation and the potential pros and cons. They were reassured of their right to withdraw without repercussions, promoting a comfortable environment. A pilot study was conducted to assess feasibility, applicability, resource allocation, time requirements, cost implications, quality enhancement, and the efficiency of the designed methodology, ensuring it would generate the desired results from the planned research study. It involved selecting 10 RNs from different pediatric hospital departments in Riyadh city who met the exact eligibility requirements. It is crucial to note that the pediatric RNs involved in the pilot study were not part of the main study. The high level of collaboration among participants led to a 100% response rate. Filling out the surveys took around 10–15 min, as the questions were written clearly and concisely and did not require any changes.

### 3.3. Instrumentation

It consists of four sections: the demographic part collects information on participants’ age, gender, nationality, marital status, work unit, experience, family structure, and living environment; the second part is the Self‐Compassion Scale (SCS) developed by Neff [36]; this 26‐item scale employs a five‐point Likert response format. It includes positive self‐compassion (self‐kindness, common humanity, and mindfulness) and negative (self‐judgment, isolation, and over‐identification). Scores are adjusted to reflect overall self‐compassion. The third part is the Compassion Competence Scale (CCS), created by Lee and Seomun. The 17‐item CCS assesses compassion competence in nursing across three subdomains: communication, sensitivity, and insight, using a five‐point scale. The total mean score indicates overall compassion competence. The last part is the Caring Behaviors Inventory Version 16 (CBI‐16), which evaluates nurses’ caring practices based on Jean Watson’s theory of human caring. Originally a 75‐item scale, the CBI has been psychometrically refined into a 16‐item inventory that assesses dimensions of respectful communication, presence, and professional skills using a six‐point Likert scale. The total score ranges from 16 to 96, with higher scores reflecting better perceptions of caring behaviors. Neff [36] established the validity and reliability of the SCS with a Cronbach’s alpha of 0.92. Subscale alphas were self‐kindness (0.78), self‐judgment (0.77), common humanity (0.80), isolation (0.79), mindfulness (0.75), and over‐identification (0.81). The SCS was culturally translated into Arabic and demonstrated psychometric properties in a study with Saudi nursing students. An exploratory factor analysis (EFA) revealed six dimensions, accounting for 64.1% of the variance, and reported Cronbach’s alpha ranging from 0.76 to 0.85, yielding an overall coefficient of 0.86; the intraclass correlation coefficient (ICC) for the SCS‐A was 0.81 [37]. The CCS showed strong validity and reliability with a Cronbach’s alpha of 0.91. Subscale alphas were communication (0.88), sensitivity (0.77), and insight (0.73) [38]. The CCS was translated into Arabic and tested with Saudi nursing students and interns. EFA indicated a three‐factor solution with 50.62% variance, and Cronbach’s alpha ranged from 0.739 to 0.797, with an overall coefficient of 0.806; ICC for the CCS‐A was 0.84 [39]. Based on Watson’s theory, the CBI‐16 measures patients’ perceptions of nursing care. Factor analysis identified one factor with 58.0% variance and excellent internal consistency (*α* = 0.95) [40]. The CBI‐16 was culturally adapted and translated into Arabic, yielding a single‐factor solution with 67% variance and a Cronbach’s alpha of 0.961 [11]. This study reported Cronbach’s alpha values of 0.83 for the SCS, 0.92 for the CCS, and 0.93 for the CBI‐16. While Arabic validation studies supported regional reliability, the questionnaire used here was entirely in English.

### 3.4. Data Analysis Methods

All survey questions in the Google form were mandatory, ensuring no missing data. Data confidentiality was maintained by storing all files on a password‐protected computer accessible only to the research team. Data were initially cleaned and structured in Microsoft Excel and subsequently coded and analyzed using IBM SPSS Statistics (Version 29). Descriptive statistics (means and standard deviations) were generated for self‐compassion, compassion competence, and caring behavior. Pearson’s correlation coefficients were used to examine associations among these variables. With a sample of 202 nurses, the score distributions approximated normality, allowing the subscales to be treated as continuous variables. Independent samples *t*‐tests and one‐way ANOVA were used to evaluate differences across demographic categories. When assumptions of homogeneity were met, Tukey’s HSD was used for post hoc comparisons; otherwise, Dunnett’s C was employed. These analyses adhered to assumptions of normality, independence, and homoscedasticity [33]. Multiple regression analyses assessed the impact of compassion competence on caring behavior, and a moderation analysis tested whether self‐compassion modified this relationship. For assumption testing, diagnostic checks were performed before analyzing the moderation results. Residuals showed approximate normality, with slight tail deviations evident in histograms and Q–Q plots, and the Shapiro–Wilk test (*W* = 95, *p* < 0.001) indicated nonnormality.

The Breusch–Pagan test revealed heteroscedasticity (*χ* ^2^ = 54.48, *p* < 0.001); thus, the regression models were reestimated with robust standard errors. This adjustment did not impact the significance of the predictors. The Durbin–Watson statistic of 1.97 supported the assumption of independence of errors. Variance inflation factors were high (48.13–68.93), indicating multicollinearity due to the interaction term. Nonetheless, the moderation effect remained nonsignificant, with no change in interpretation. Cook’s distance identified 10 influential points; a sensitivity analysis showed that excluding them did not substantially alter the regression coefficients. Overall, these diagnostic results confirm the reliability and stability of the final model.

## 4. Results

The questionnaire was sent online to 276 nurses working in pediatric units; 202 agreed to participate, resulting in a response rate of 73% of the total sample. The sample consisted predominantly of females (97%, 196), with only 3% (6) males. Most participants were married (54%, 109) or single (44.1%, 89), with a small percentage identifying as other (2%, 4). Regarding nationality, 63.9% (129) were non‐Saudi, while 36.1% (73) were Saudi nationals. The majority of respondents came from nuclear families (58.4%, 118) and extended families (14.6%, 84), and most of them lived in urban areas (59.9%, 121) and rural areas (40.1%, 81). Age‐wise, 45.5% (92) were between 31 and 40 years, followed by 38.6% (78) under 30 years, 12.4% (25) between 41 and 50 years, and 3.5% (7) over 50 years. Regarding work experience, 38.6% (78) had 6–10 years, 32.7% (66) had more than 10 years, 18.3% (37) had 3–5 years, and 10.4% (21) had 1–2 years. The work areas included Pediatric/Surgical Ward (34.7%, 70), PICU (20.3%, 41), Emergency Department (16.8%, 34), NICU (15.8%, 32), and Outpatient Clinics (12.4%, 25).

### 4.1. Self‐Compassion, Compassion Competence, and Caring Behavior of Pediatric Nurses

The assessment of self‐compassion yielded the following results. The self‐kindness subscale had a high mean score (*M* = 3.86), with the highest item being “I try to be loving toward myself when I’m feeling emotional pain” (*M* = 4.08) and the lowest being “I’m tolerant of my flaws” (*M* = 3.71). The common humanity subscale also scored high (*M* = 3.65), with the highest item being “I try to see my failings as part of the human condition” (*M* = 3.93) and the lowest being “When I feel inadequate, I remind myself that these feelings are shared” (*M* = 3.43). The self‐judgment subscale showed moderate self‐judgment (*M* = 2.80), with “When times are really difficult, I tend to be tough on myself” (*M* = 3.03) being the highest and “I’m disapproving and judgmental about my flaws” (*M* = 2.65) being the lowest. Mindfulness also indicated moderate levels (*M* = 2.88), with “When something upsets me, I try to keep my emotions in balance” (*M* = 4.16) as the highest and “When I’m down, I approach my feelings with curiosity” (*M* = 3.66) as the lowest. The isolation subscale demonstrated moderate isolation (*M* = 2.76), with “When I fail, I tend to feel alone” (*M* = 2.92) being the highest and “Thinking about my inadequacies makes me feel cut off” (*M* = 2.53) being the lowest. Over‐identification showed moderate (*M* = 2.95), with “When something upsets me, I get carried away with my feelings” (*M* = 3.21) being the highest and “When I feel down, I obsess over what’s wrong” (*M* = 2.72) being the lowest. Overall, self‐compassion scored high (*M* = 3.48), with mindfulness leading (*M* = 3.88), followed by self‐kindness (*M* = 3.86) and common humanity (*M* = 3.65). Among negative subscales, over‐identification was the highest (*M* = 2.92), followed by self‐judgment (*M* = 2.80) and isolation (*M* = 2.76) (see Table 1).

**Table 1:** Mean and standard deviation of self‐compassion, compassion competence, and caring behavior for participants.

Subscale	Mean	SD
Self‐compassion		
Self‐kindness	3.86	0.78
Common humanity	3.65	0.81
Mindfulness	3.88	0.81
Self‐judgment	2.80	0.81
Isolation	2.76	0.91
Over‐identification	2.92	0.86
Total scale	3.48	0.52
Compassion competence		
Communication	4.02	0.62
Sensitivity	4.17	0.68
Insight	4.00	0.74
Total scale	4.06	0.59
Caring behavior		
Respectful	5.40	0.71
Connectedness	5.27	0.77
Knowledge and skills	5.45	0.73
Assurance	5.43	0.68
Total sum score ± SD	86.33	10.31

For compassion competence, results indicated high communication (*M* = 4.02) with the highest item being “I am aware of how to communicate with patients to encourage them” (*M* = 4.17) and the lowest being “When communicating with patients, I respond to them with proper nonverbal presentations” (*M* = 3.86). The sensitivity subscale also showed high levels (*M* = 4.17), with “I am careful with my speech and behavior to avoid hurting my patients’ feelings” (*M* = 4.32) as the highest and “I am tolerant of others’ opinions” (*M* = 3.90) as the lowest. For the insight subscale, the overall score was high (*M* = 4.00), with “I look after patients without being influenced by personally challenging situations” and “I can empathize well with patients’ difficulties” both at *M* = 4.01 and the lowest being “I am intuitive about patients because of my diverse clinical experience” (*M* = 3.97). The overall CCS showed high competence (*M* = 4.06), with sensitivity being the highest subscale (*M* = 4.17), followed by communication (*M* = 4.02) and insight (*M* = 4.00) (see Table 1).

Regarding caring behavior, results revealed a high respect score (*M* = 5.40), with “Treating the patient as an individual” (*M* = 5.48) being the highest and “Being empathetic or identifying with the patient” (*M* = 5.29) being the lowest. The connectedness subscale also showed high levels (*M* = 5.27), with “Giving instruction or teaching the patient” (*M* = 5.38) being the highest, while “Spending time with the patient” (*M* = 5.06) ranked lowest. The knowledge and skills subscale exhibited high competence (*M* = 5.45), with the highest score for “Treating the patient’s information confidentially” (*M* = 5.62) and the lowest for “Meeting patients’ stated and unstated needs” (*M* = 5.30). The assurance subscale achieved a score of 5.43, with “Giving patients their treatments and medications on time” being the highest (*M* = 5.63) and “Returning to the patient voluntarily” being the lowest (*M* = 5.28). Overall, caring behavior scored high (*M* = 86.33), with the highest subscale being knowledge and skills (*M* = 5.45), followed by assurance (*M* = 5.43), respect (*M* = 5.40), and connectedness (*M* = 5.27) (see Table 1).

### 4.2. Relationships Between Study Variables

Table 2 presents moderate positive correlations between self‐kindness and common humanity (*r* = 0.427, *p* < 0.001) and mindfulness (*r* = 0.693, *p* < 0.001), as well as a weak negative correlation with isolation (*r* = −0.177, *p* = 0.012). Common humanity correlates moderately with mindfulness (*r* = 0.431, *p* < 0.001) and weakly with self‐judgment (*r* = 0.249, *p* < 0.001). Mindfulness shows a weak negative correlation with isolation (*r* = −0.224, *p* < 0.001). Self‐judgment correlates positively with isolation (*r* = 0.720, *p* < 0.001) and over‐identification (*r* = 0.672, *p* < 0.001) and weakly positively with itself (*r* = 0.682, *p* < 0.001). For the compassion competence subscales, moderate positive correlations are observed between communication and sensitivity (*r* = 0.707, *p* < 0.001), communication and insight (*r* = 0.636, *p* < 0.001), and sensitivity and insight (*r* = 0.720, *p* < 0.001). There are moderate positive correlations between self‐compassion and compassion competence (*r* = 0.285, *p* < 0.001), a weak positive correlation with caring behavior (*r* = 0.183, *p* = 0.009), and a moderate positive correlation between compassion competence and caring behavior (*r* = 0.397, *p* < 0.001).

**Table 2:** Correlation matrix between self‐compassion, compassion competence, and caring behavior and their subscales.

Scales/subscales		1	2	3	4	5	6	7	8	9	10	11	12	13	14	15	16
1. Self‐kindness	*r*																
	*p*																

2. Common humanity	*r*	0.427															
	*p*	0.000															

3. Mindfulness	*r*	0.693	0.431														
	*p*	0.000	0.000														

4. Self‐judgment	*r*	−0.137	0.249	−0.118													
	*p*	0.052	0.000	0.095													

5. Isolation	*r*	−0.177	0.100	−0.224	0.720												
	*p*	0.012	0.158	0.001	0.000												

6. Over‐identification	*r*	−0.031	0.119	−0.107	0.672	0.682											
	*p*	0.663	0.091	0.130	0.000	0.000											

7. Total	*r*	0.654	0.334	0.667	−0.676	−0.743	−0.649										
	*p*	0.000	0.000	0.000	0.000	0.000	0.000										

8. Communication	*r*	0.306	0.161	0.173	−0.132	−0.163	0.014	0.249									
	*p*	0.000	0.022	0.014	0.061	0.021	0.847	0.000									

9. Sensitivity	*r*	0.263	0.161	0.177	−0.096	−0.199	−0.053	0.253	0.707								
	*p*	0.000	0.022	0.012	0.174	0.005	0.457	0.000	0.000								

10. Insight	*r*	0.226	0.205	0.157	−0.152	−0.186	−0.067	0.265	0.636	0.720							
	*p*	0.001	0.003	0.026	0.030	0.008	0.342	0.000	0.000	0.000							

11. Total	*r*	0.305	0.193	0.190	−0.142	−0.201	−0.031	0.285	0.916	0.895	0.848						
	*p*	0.000	0.006	0.007	0.044	0.004	0.665	0.000	0.000	0.000	0.000						

12. Respectful	*r*	0.259	0.229	0.249	−0.021	0.025	0.038	0.180	0.326	0.340	0.380	0.386					
	*p*	0.000	0.001	0.000	0.763	0.728	0.595	0.010	0.000	0.000	0.000	0.000					

13. Connectedness	*r*	0.211	0.105	0.228	−0.065	−0.109	−0.020	0.195	0.273	0.325	0.367	0.351	0.782				
	*p*	0.003	0.138	0.001	0.358	0.123	0.778	0.005	0.000	0.000	0.000	0.000	0.000				

14. Knowledge	*r*	0.222	0.132	0.212	−0.019	−0.057	0.035	0.159	0.289	0.359	0.367	0.370	0.735	0.763			
	*p*	0.002	0.060	0.002	0.785	0.424	0.616	0.024	0.000	0.000	0.000	0.000	0.000	0.000			

15. Assurance	*r*	0.222	0.179	0.250	0.027	0.000	0.092	0.136	0.304	0.259	0.305	0.326	0.748	0.691	0.747		
	*p*	0.001	0.011	0.000	0.698	0.996	0.192	0.054	0.000	0.000	0.000	0.000	0.000	0.000	0.000		

16. Total	*r*	0.255	0.183	0.262	−0.017	−0.034	0.046	0.183	0.333	0.353	0.391	0.397	0.905	0.883	0.902	0.902	
	*p*	0.000	0.009	0.000	0.812	0.634	0.512	0.009	0.000	0.000	0.000	0.000	0.000	0.000	0.000	0.000	

*Note:* r = Pearson’s correlation coefficient; *p* = significance level.

### 4.3. The Difference in the Scales due to Demographic Characteristics

Table 3 shows a significant difference in self‐compassion levels between Saudi and non‐Saudi participants (*t* = −2.040, *p* = 0.043), with non‐Saudis exhibiting higher levels. No differences in other demographics were significant. Age also revealed a significant difference in compassion competence (*F* = 2.730, *p* = 0.045), but post hoc tests did not identify specific age group differences. Years of experience significantly affected compassion competence (*F* = 2.78, *p* = 0.042), with those with over 10 years of experience reporting higher levels than those with 1–2 years; no other groups differed. Work area differences were noted (*F* = 3.047, *p* = 0.08), with outpatient clinic staff having lower compassion competence than NICU or ER personnel, but no other differences emerged. Significant differences in caring behavior were observed based on gender (*t* = 3.39, *p* = 0.008), with males showing higher levels; marital status (*F* = 5.529, *p* = 0.005), where married individuals displayed more caring behavior; nationality (*t* = −2.836, *p* = 0.005), with non‐Saudis again higher; and family type (*t* = 2.626, *p* = 0.009), showing nuclear families exhibiting greater levels than extended families. Work area differences were significant (*F* = 3.296, *p* = 0.012), with outpatient clinic staff exhibiting lower levels of caring behavior than those in pediatric/surgical wards or ERs, but no other demographic differences were found.

**Table 3:** Statistical tests between self‐compassion, compassion competence, and caring behavior of participants.

Variables	Self‐compassion	Compassion competence	Caring behavior
Gender			
Male	3.14	4.14	91
Female	3.50	4.06	86.19
*T* (*p*)	−1.651 (0.10)	0.324 (0.746)	3.39 (0.008)
Marital status			
Single	3.48	3.95	84.09^b^
Married	3.50	4.15	88.42^a^
Other (divorced/widow)	3.19	3.91	79.25^a,b^
*F* (*p*)	0.690 (0.503)	2.897 (0.058)	5.529 (0.005)
Nationality			
Saudi	3.39	3.97	83.64
Non‐Saudi	3.54	4.11	87.85
*t* (*p*)	−2.040 (0.043)	−1.801 (0.073)	−2.836 (0.005)
Family type			
Nuclear	3.57	4.10	87.92
Extended	3.42	4.00	84.11
*t* (*p*)	−2.085 (0.038)	1.257 (0.210)	2.626 (0.009)
Residence			
Rural	3.49	4.07	85.00
Urban	3.48	4.06	87.22
*t* (*p*)	0.026 (0.979)	0.104 (0.918)	−1.507 (0.133)
Age			
< 30 years	3.36	3.93	84.47
31–40 years	3.55	4.11	86.88
41–50 years	3.58	4.21	89.04
> 50 years	3.57	4.35	90.14
*F* (*p*)	2.41 (0.068)	2.730 (0.045)	1.85 (0.140)
Experience			
1–2 years	3.28	3.78^b^	83.62
3–5 years	3.52	3.98^a,b^	87.08
6–10 years	3.53	4.07^a,b^	84.99
> 10 years	3.48	4.18^a^	88.36
*F* (*p*)	1.338 (0.263)	2.780 (0.042)	1.87 (0.136)
Work area			
Pediatric/surgical ward	3.60	4.01^a,b^	87.01^a^
Outpatient clinics	3.53	3.76^b^	80.00^b^
Emergency department	3.44	4.23^a^	89.26^a^
PICU	3.36	4.06^a,b^	86.15^a,b^
NICU	3.40	4.22^a^	86.91^a,b^
*F* (*p*)	1.848 (0.121)	3.047 (0.018)	3.296 (0.012)

*Note:* Post hoc test (Tukey) (Dunnett’s C).

^a,b^Different letters for significant differences between groups.

### 4.4. The Moderation of Self‐Compassion Between the Relationship of Compassion Competence and Caring Behavior of Pediatric RNs

Multiple regression was conducted to assess the moderation effect and examine the relationship between compassion competence (CCS), self‐compassion (SCS), and their interaction (moderation) on caring behavior (CBI). According to Table 4, the overall regression model examining the effects of compassion competence (CCS), self‐compassion (SCS), and their interaction (moderation term) on caring behavior (CBI) was statistically significant (*F* = 13.74, *p* < 0.001), the results indicated there was a significant impact of compassion competence level on the caring behavior level among pediatric RNs (*t* = 2.225, *p* = 0.027), where for one unit increase in compassion competence, caring behavior level increases by 1.109 (*β* = 1.109), but the impact of self‐compassion and its interaction with compassion competence on caring behavior was not significant. The interaction between compassion, competence, and self‐compassion was not significant, indicating that self‐compassion did not moderate this relationship in the present sample. When demographic factors were included as covariates in a sensitivity analysis, the relationship between compassion competence and caring behavior remained significant, and the moderation effect of self‐compassion remained nonsignificant. These results demonstrate that the core findings were stable and not dependent on demographic characteristics within the sample.

**Table 4:** The moderation of self‐compassion between the relationship of compassion competence and caring behavior.

Predictors	*β*	*t*	*p* value
Self‐compassion	0.852	1.616	0.108
Compassion competence	1.109	2.225	0.027
Moderation effect	−1.214	−1.485	0.139

*Note:* Model (*F* = 13.74, *p* < 0.001).

## 5. Discussion

### 5.1. The Finding of Self‐Compassion Subscales of Pediatric RNs

The study demonstrated that pediatric RNs reported higher self‐compassion levels, with mean scores exceeding the midpoint across the positive subscales—self‐kindness, common humanity, and mindfulness—indicating an overall compassionate self‐orientation. High self‐compassion is especially important in high‐stress clinical environments, such as pediatric settings, where emotional labor is substantial. Consistent with previous literature, self‐compassion serves as a protective psychological resource that enhances emotional resilience, enables nurses to navigate morally complex situations, and supports their overall well‐being [41, 42].

Importantly, pediatric RNs scored lower on negative subscales—self‐judgment, isolation, and over‐identification—suggesting stronger coping mechanisms and reduced vulnerability to negative self‐appraisals. This aligns with regional evidence showing that many nurses in Middle Eastern cultures draw upon interpersonal connectedness, religious values, and collectivist coping to regulate stress, which may naturally reinforce elements of common humanity and emotional balance [43].

Saudi Arabia’s cultural and religious context may further contribute to this finding. Islamic ethical principles emphasize compassion, mercy (*rahma*), and patience, values deeply embedded in Saudi nursing education and professional identity. These values may enhance nurses’ ability to maintain self‐kindness and emotional grounding when caring for vulnerable children and families. Pediatric nurses often care for critically ill children, requiring advanced empathy and sustained emotional engagement, which supports the development of self‐compassion skills over time [44].

Similarly, international and regional studies show that nurses with higher self‐compassion experience improved resilience, lower burnout, and more stable emotional regulation [15, 45]. Altogether, the findings indicate that pediatric RNs in Saudi Arabia possess moderate to high self‐compassion, an essential factor for sustaining compassionate care and personal well‐being.

### 5.2. The Finding of the Compassion Competence Subscales of Pediatric RNs

The study revealed that pediatric RNs have high compassion competence, with sensitivity scoring the highest. This demonstrates strong emotional sensitivity, marked by increased empathic awareness and responsiveness to patient needs, which are crucial in pediatric care, as it involves interpreting nonverbal cues, managing fear, addressing developmental concerns, and alleviating parental anxiety. High scores in communication and insight further demonstrate pediatric nurses’ ability to convey compassion effectively and understand patient perspectives.

These findings mirror international research [46, 47] and align with Saudi studies that highlight the professional expectation that nurses demonstrate empathy, emotional warmth, and patient‐centered care. Cultural expectations in Saudi Arabia encourage compassionate caregiving as a religious, ethical, and social duty, potentially enhancing nurses’ sensitivity and communication with children and families [8, 11]. Moreover, high compassion competence in this study aligns with results from oncology and intensive care settings in the region [48, 49]. However, the findings contrast with those of Arkan et al. [50], who reported moderate levels among pediatric nurses, suggesting that Saudi pediatric nurses may benefit from stronger institutional support, greater cultural emphasis on caring, or better access to training that reinforces compassionate communication. Given the increasing complexity of pediatric care in Saudi Arabia, nursing education programs and hospital systems should continue to prioritize compassion, competence training, communication skills, and reflective practice, as recommended by the global literature [14, 51].

### 5.3. The Finding of the Caring Behavior Subscales of Pediatric RNs

Pediatric RNs demonstrated significantly high caring behavior scores. This outcome is likely influenced by Saudi Arabia’s cultural and religious framework, in which caring for the sick is regarded as an ethical and spiritual duty grounded in Islamic values. Caring behaviors, such as empathy, generosity, and attentiveness, are culturally reinforced and expected in family‐centered societies like Saudi Arabia [39].

Within Saudi nursing education, curricula emphasize integrating technical competence with emotional expressions of care, aligning with the Ministry of Health’s patient‐centered care model and national nursing competencies [11, 52]. This cultural and educational alignment contributes to the consistently high caring behavior observed among Saudi nurses. Regional studies also support these findings: Nurses in intensive care settings in Turkey and Saudi Arabia demonstrated high levels of perceived care quality and caring behaviors [49, 53]. Pediatric care further amplifies the need for emotional protection, clear communication, and family support, central tenets of caring behavior in Middle Eastern contexts.

### 5.4. The Findings of Demographic Variables and Study Variables

The study found no significant gender differences in self‐compassion, although male participants demonstrated higher compassion competence and caring behaviors. The literature remains mixed: Some studies report higher self‐compassion among males [54], while others report lower levels among women [55]. Importantly, the present study’s gender findings must be interpreted with caution due to the markedly unequal distribution of participants, with males representing a very small proportion of the sample. This imbalance limits the reliability of gender comparisons and prevents generalization of results to male pediatric nurses. However, these findings should be interpreted with caution due to the extremely small number of male nurses in the sample. Gender disparities in Saudi nursing continue to be shaped by cultural norms, workforce trends, and the development of professional identity. Nursing in Saudi Arabia remains predominantly female, though national reforms are increasing male participation. Cultural expectations may also influence men’s expression of compassion competence, aligning with social role theories in Middle Eastern societies that encourage men to demonstrate strength, decisiveness, and protective caring behaviors [56]. Marital status did not significantly impact self‐compassion, contrary to earlier findings linking unmarried status to lower compassion fatigue [57]. Nationality differences indicated higher self‐compassion and caring among non‐Saudi nurses, consistent with the literature, which suggests that expatriate nurses often draw on diverse cultural backgrounds, coping strategies, and professional experiences [11]. Cultural norms strongly shape perceptions of self‐compassion across societies [58, 59]. The study revealed that nurses from nuclear families displayed higher self‐compassion and caring behaviors. In Saudi Arabia, extended family systems may increase caregiving responsibilities outside work, contributing to emotional strain and reduced capacity for self‐care. Conversely, nuclear family settings may afford greater autonomy and space for self‐reflection [60].

Additionally, age and years of experience were significant predictors of compassion competence, consistent with research showing that compassion deepens with maturity, exposure to patient suffering, and improved emotional regulation [61]. Experienced nurses also demonstrate stronger coping strategies and lower susceptibility to compassion fatigue [62]. Furthermore, the outpatient pediatric nurses displayed lower compassion competence and caring behavior than those in intensive care units or emergency departments. Environmental factors—such as acuity level, patient turnover, and team support—likely shape these differences. In Saudi settings, NICU and ER environments provide stronger interprofessional collaboration and clinical engagement, which may reinforce compassionate practice [63].

### 5.5. The Moderating Effect of Self‐Compassion on the Relationship Between Compassion Competence and Caring Behavior

The moderating analysis demonstrated that self‐compassion did not significantly alter the relationship between compassion competence and caring behavior. While self‐compassion was positively associated with caring behavior, its effect was not strong enough to influence the interaction between the constructs.

This diverges from studies that found that self‐compassion enhances caring behavior [24] but aligns with research suggesting that compassion competence itself is the most influential determinant of caring behavior among nurses in high‐stakes clinical settings [17, 64]. In Saudi pediatric practice, compassion competence may be strengthened through cultural expectations, clinical exposure, and spiritual motivations, reducing the relative contribution of self‐compassion as a moderator. Although not statistically significant, self‐compassion remains clinically relevant: It reduces burnout, improves emotional resilience, and supports sustained engagement in caring practices [65, 66]. Therefore, interventions that enhance both compassion competence and self‐compassion may yield synergistic benefits for pediatric nurses.

This study has several limitations. Although validated Arabic versions of the study instruments were used, psychometric properties were not reassessed in this sample, as scale validation was beyond the study’s scope. The cross‐sectional design also limits causal interpretation. The sample was predominantly female, reflecting the pediatric nursing workforce in Saudi Arabia but limiting the generalizability of gender‐related findings to male nurses. Future research with more balanced gender representation is necessary to draw robust conclusions about potential gender differences in self‐compassion, compassion competence, and caring behavior. While regression diagnostics supported model stability, issues such as heteroscedasticity and influential observations warrant cautious interpretation. Finally, reliance on self‐reported data may introduce response bias. Future research should incorporate longitudinal designs, diverse sampling, and renewed psychometric evaluation to strengthen generalizability.

## 6. Conclusion

The study revealed high self‐compassion among pediatric RNs in Riyadh, Saudi Arabia, with mindfulness, self‐kindness, and shared humanity as the most prominent positive subscales. Conversely, over‐identification, self‐judgment, and isolation were noted among the negative subscales. Moderate positive correlations were identified between self‐judgment and isolation and between self‐judgment and over‐identification. Significant differences in self‐compassion levels were observed between Saudi and non‐Saudi RNs, whereas no significant differences were found in other demographic variables. Additionally, a high level of compassion competence was reported, with sensitivity, communication, and insight being the highest subscales. Moderate positive correlations were observed between communication and sensitivity, communication and insight, and sensitivity and insight. Compassion competence varied significantly based on age, years of experience, and work area. Gender, marital status, nationality, family type, and work area also demonstrated significant disparities in caring behavior. Correlations included a moderate positive relationship between self‐compassion and compassion competence, a weak positive correlation between self‐compassion and caring behavior, and a moderate positive correlation between compassion competence and caring behavior. The effect of compassion competence on caring behavior was significant, whereas self‐compassion and the interaction between self‐compassion and compassion competence were not significant predictors. This study highlights the need for healthcare organizations and nurse managers to recognize individual and clinical differences among RNs when creating interventions to foster self‐compassion and caring behaviors. By emphasizing compassion training, healthcare institutions can foster a more empathetic environment that enhances communication, improves health outcomes, and builds trust between patients and providers. Strategic investment in compassion competence not only at the frontline but also through managerial leadership yields benefits across the healthcare system, improving patient and family experiences while supporting workforce well‐being and professional performance.

## Author Contributions

Meaad T. Alanazi and Manal F. Alharbi contributed equally to this research in conceptualization, data curation, formal analysis, funding acquisition, investigation, methodology, project administration, resources, software, supervision, validation, visualization, writing–original draft, and writing–review and editing.

## Funding

This study was funded by King Saud University, Riyadh, Saudi Arabia.

## Ethics Statement

IRB approval was obtained from King Khalid University Hospital and King Saud University for Project No. E‐24‐8534. An informed consent section states that by proceeding, participants agree to participate, serving as their written consent. Following this, participants completed a questionnaire without providing any identifiable personal information. Email approval was granted for using SCS, CCS, and the CBI‐16 tool. Ethical guidelines from the Belmont Report (1979) and the Declaration of Helsinki (1964) were followed to ensure participants’ rights and safety.

## Conflicts of Interest

The authors declare no conflicts of interest.

## Data Availability

The data that support the findings of this study are available from the corresponding author upon reasonable request.
